# Preparation of Modified Films with Protein from Grouper Fish

**DOI:** 10.1155/2016/3926847

**Published:** 2016-08-11

**Authors:** M. A. Valdivia-López, A. Tecante, S. Granados-Navarrete, C. Martínez-García

**Affiliations:** Departamento de Alimentos y Biotecnología, Facultad de Química, Universidad Nacional Autónoma de México, 04510 Ciudad de México, Mexico

## Abstract

A protein concentrate (PC) was obtained from Grouper fish skin and it was used to prepare films with different amounts of sorbitol and glycerol as plasticizers. The best performing films regarding resistance were then modified with various concentrations of CaCl_2_, CaSO_4_ (calcium salts), and glucono-*δ*-lactone (GDL) with the purpose of improving their mechanical and barrier properties. These films were characterized by determining their mechanical properties and permeability to water vapor and oxygen. Formulations with 5% (w/v) protein and 75% sorbitol and 4% (w/v) protein with a mixture of 15% glycerol and 15% sorbitol produced adequate films. Calcium salts and GDL increased the tensile fracture stress but reduced the fracture strain and decreased water vapor permeability compared with control films. The films prepared represent an attractive alternative for being used as food packaging materials.

## 1. Introduction

Biodegradable films are an attractive development for the food industry. Their use is associated with the broad range of properties they possess. Such properties are helpful to keep food in optimum conditions during transport and storage and constitute an interesting answer to the demand of consumers for higher quality and long-shelf life products, while reducing disposable packaging material and increasing recyclability [1]. The extensive list of biodegradable film ingredients available allows targeting a broad range of potential functional properties [2].

A biodegradable film is defined as a thin continuous layer made from biodegradable materials [3], that is, materials that can be degraded by enzyme action of living organisms, such as bacteria, yeast, and fungi [4]. Some critical conditions such as abundance and availability of raw materials are needed to make the production of biodegradable polymers feasible. Protein is one resource that meets these characteristics [5]. Proteins, polysaccharides, and lipids have been used as film-forming materials. However, proteins have been widely chosen because they are abundant, are available in plant and animal sources, and form stable networks [6]. Also, protein films are better than those prepared with polysaccharides because proteins are composed of 20 different amino acids, and they have a particular structure which offers a broad range of functional properties [7].

Fish skin is a good source of inexpensive collagen, which is the main support protein that constitutes the structures of the body of animals, vertebrates, and invertebrates and is concentrated in specialized connective tissues: skin, tendon, and bone [8]. Castañeda [9] studied the properties of fish skin proteins demonstrating that it is useful to form biodegradable films. The results obtained revealed the films with 5% protein concentrate (PC) and 75% sorbitol (plasticizer) to present the best structural characteristics, and although they exhibited acceptable mechanical properties, their barrier properties to moisture migration were not good. Water vapor loss is one of the more severe problems in food preservation, and it can cause adverse effects on texture, nutritive value, scalability, and integrity of food products.

Several properties of fish skin films, such as mechanical properties, permeability, light absorption, transparency, antimicrobial activity, and antioxidant ability, are influenced by the addition of active substances [10]. For example, Park and collaborators [11] modified biodegradable soy films, adding CaCl_2_ and CaSO_4_ (calcium salts) and glucono-*δ*-lactone (GDL). They concluded that calcium salts and GDL reduced the water vapor permeability and improved their mechanical properties. Also, Zactiti and Kieckbusch [12] added calcium salts to alginate films and observed lower elongation, higher tensile strength, and a considerable reduction in water solubility and water vapor permeability.

In Mexico around 11300 tons of Grouper fish (*Epinephelus marginatus*) are captured per year [13], signifying one of the major domestic fisheries. Grouper is consumed as fresh and frozen fillet, and the skin, which is about 10% of the weight of the fish, is not used as a commercial product; this means that about 1300 tons of skin of this fishery are discarded. Fish skins are a major by-product of fishing and aquaculture. Thus, the fish skin could provide a valuable source of protein [14]. Many fish skin films produced from fish-processing coproducts have been studied, such as the skins of cuttlefish [15], blue shark [14], bigeye snapper, and brownstripe red snapper [6], showing, in general, poor mechanical properties and high water vapor permeability which are the main drawbacks for applications. Therefore, the aim of the present work was to study film formation from collagen fractions extracted from the skin of Grouper fish (*Epinephelus marginatus*) using plasticizers and different concentrations of calcium salts and glucono-*δ*-lactone and to evaluate the effect on its mechanical and barrier properties.

## 2. Materials and Methods

### 2.1. Compositional Analysis of Grouper Fish Skin

The skin of Grouper fish (*Epinephelus marginatus*) was obtained from a local market in Mexico City. The skin of three different batches, each one with three replicas, was analyzed for moisture, fat, and total crude protein content. Assays were done using AOAC methods [16]: moisture in a vacuum oven (931.04); fat, goldfish (920.85); and total crude protein, Kjeldahl (981.10).

### 2.2. Protein Concentrate

Protein was extracted from fresh fish skin according to the procedure of Batista [17], with some modifications. The skin was cut into small pieces, soaked in 0.1 M NaOH (pH 12 ± 0.5) with skin to water ratio of 1 : 10 (w/v), respectively, and the mixture was stirred 120 min at 45°C. After this time, the suspension was centrifuged (Beckman J2 centrifuge J2-mark M2) 15 min at 4°C and 5000 rpm, and the supernatant was recovered. At this point, the protein content expressed as total crude protein, and soluble protein was determined in the supernatant, while the residue, composed of milled skin and scales, was examined for total crude protein** (**Kjeldahl AOAC 981.10). One milliliter of 10% sodium hexametaphosphate was added to 50 mL of supernatant, pH was adjusted to 2.5 with 2 M HCl, and the liquid was kept two hours in refrigeration for complete precipitation of proteins. After this, proteins were separated by centrifugation at 5000 rpm for 15 minutes. The residue constitutes the protein concentrate (PC); its amount of total crude and soluble proteins was determined and the PC pellet was frozen until further needed. This PC was used for film formation. The yield of protein extraction was determined in every one of the extracted fractions from measurements of total crude protein and soluble protein [18]. Fish skin and protein concentrate were weighed and the volume of supernatants was measured.

### 2.3. Molecular Weight of Extracted Proteins

The protein fractions in the concentrate were separated by sodium dodecylsulfate-polyacrylamide gel electrophoresis (SDS-PAGE) according to Laemmli [19]. A 10% polyacrylamide gel was used for high molecular weights (36–200 kDa Sigma Marker Sigma) and a 12% gel for low molecular weights (20–66 kDa Sigma Marker). The solution, with 1 and 2 mg/mL PC (pH 12 ± 0.1), was mixed with buffer in a 1 : 1 (v/v) ratio and heated for 3 min in boiling water. Gels were loaded with the treated sample (20 mL) and with molecular weight markers (4 mL) and were run in an electrophoresis chamber (Bio-Rad) at 100 volts for 90–120 min. After this, gels were stained overnight with Coomassie blue solution and washed with a 10 : 10 : 80% (v/v/v) methanol/acetic acid/water solution. The washed gels were scanned in a densitometer (Bio-Rad, Model GS700) and the molecular weights of the separate bands were determined with the Quantity-One software (Bio-Rad).

### 2.4. Solubility of the Protein Concentrates at Different pH

The variation of solubility with pH was determined following the method of Saeed and Cheryan [20]. The pH of twelve aqueous solutions (10 mL) with 3% (w/v) PC was adjusted (Thermo Electron Corporation, USA) to values in the range of 1.0 to 12.0 with 1.0 N HCl and NaOH. Protein solutions were stirred for 30 min. Solutions were then centrifuged (Labtronic Scientific, Model H-1650) at 2500 rpm for 15 min and the PC of the supernatant was determined using Lowry's method [18].

### 2.5. Protein Concentrate Films

Films were prepared following the method of Sobral and collaborators [21]. Five grams of protein concentrate (PC) was dispersed in 100 mL distilled water under continuous stirring and pH was adjusted to 11.5 ± 0.2 with 1.0 N NaOH. Sorbitol and glycerol were added to different films, in concentrations of 50 and 75% (w/w) referred to the total amount of protein in the PC. An additional formulation included 4% (w/v) PC, with a mixture of 15% (w/w) glycerol and 15% (w/w) sorbitol [22]. The formulations were identified as 5PC-50G, 5PC-75G, 5PC-50S, 5PC-75S, and 4PC-15G/15S, where the first part indicates the concentration of PC and the second one the concentration of plasticizer. Different solutions were stirred for 10 min and then heated to 70°C for 20 min in a water bath. After this time, the solutions were filtered and sonicated (Branson 3510, Bransonic® ultrasonic) for 15 min. Finally, 50 mL of solution was poured into Teflon-covered pans 12 cm in diameter and dried at room temperature for approximately 48 hours (Table 1).

Films were considered adequate when they were easily detached manually from the container surface, nonsticky and flexible enough to handling. Then, these selected films were modified to improve their mechanical properties, permeability to water vapor and oxygen. The modification was done by adding CaCl_2_, CaSO_4_ (calcium salts), and glucono-*δ*-lactone (GDL) in concentrations of 0.1, 0.2, and 0.3% (w/w), of the amount of total protein in the PC [11]. The salts were added after the plasticizer; a salt solution of known concentration was added depending on the amount of protein present (Table 2). Modified and unmodified films were analyzed. The latter are designated as the control.

### 2.6. Water Vapor Permeability

Water vapor permeability (WVP) was determined according to the ASTM E96-95 method [23]. This is a gravimetric procedure in which the amount of water adsorbed by anhydrous calcium chloride is determined. Acrylic cells, previously taken to constant weight, were used. A known amount of desiccant (*ca*. 35 g) was placed in the cell leaving a head space of about 1 cm. The films were fixed to the rim of the cell with a pressing ring leaving a known area for water vapor transmission. Cells were placed in a chamber with a relative air humidity of 62 ± 2% and maintained at room temperature (≈23 ± 2°C). The increment of mass and temperature of each cell was recorded every 24 h for five days. Determinations were done in triplicate.

### 2.7. Oxygen Permeability

Oxygen permeability was determined in a stainless steel cell (CSI-135 Permeability Tester) according to the ASTM D1434-82 method [24]. The oxygen transmission coefficient was obtained by monitoring the change in volume generated by the transfer of oxygen through the film as a result of an applied differential gas pressure. The cell was operated at a manometric pressure of 4 psi (27571 Pa), 293.25 K, and a barometric pressure of 77994 Pa.

### 2.8. Mechanical Resistance

Before testing the films for mechanical resistance they were first conditioned at 62 ± 2% relative humidity and 23 ± 2°C for 48 h. Relative humidity was generated with a saturated solution of Mg(NO_3_)_2_·6H_2_O and measured with a hygrometer (Oakton, Japón). Measurements were made with a testing machine (Sintech 1/S, MTS, USA) using a load cell of 100 N according to the ASTM D 882 method [25]. Film strips 8 cm long and 1 cm wide were examined. Their average thickness was measured with a micrometer (Mitutoyo, Japan) on both ends and in the middle of the strip. Strips were stretched at 250 mm/min until they broke up. Force-time data were transformed into true stress, (1) *σ*_*T*_, and Hencky strain, (2) *ε*_*H*_. Young's modulus was determined from the slope of the linear portion of *σ*_*T*_ versus *ε*_*H*_ plot:

(1) True stress (*σ*_*T*_): *σ*_*T*_ = *σ*(1 + *e*),
(2) Hencky (*ε*_*H*_): *ε*_*H*_ = ln⁡(1 + *e*).

In these equations *σ* is the nominal stress and *e* is the Cauchy strain.

### 2.9. Statistical Analysis

All experiments were carried out in triplicate. Statistics on an entirely randomized design were determined with the SPSS 10.0 for Windows procedure. Differences were considered to be statistically significant at *p* < 0.05.

## 3. Results and Discussion

### 3.1. Yield of Protein Concentrate

The extraction process did not include a purification step to obtain an entirely pure protein concentrate. Hence, yields of total protein, 63.81%, and soluble protein, 45.33%, in the concentrate were small. Impurities in the concentrate can include salts normally present in the skin and sodium hexametaphosphate used to make precipitation easier; such impurities were possibly solubilized and precipitated together with proteins. The average composition of the three different batches was on a dry basis: 65.21 ± 2.85% moisture, 6.3 ± 0.18% fat, and 76.51 ± 4.54% total crude protein.

### 3.2. Molecular Weight of Protein Fractions

**Figure 1:**
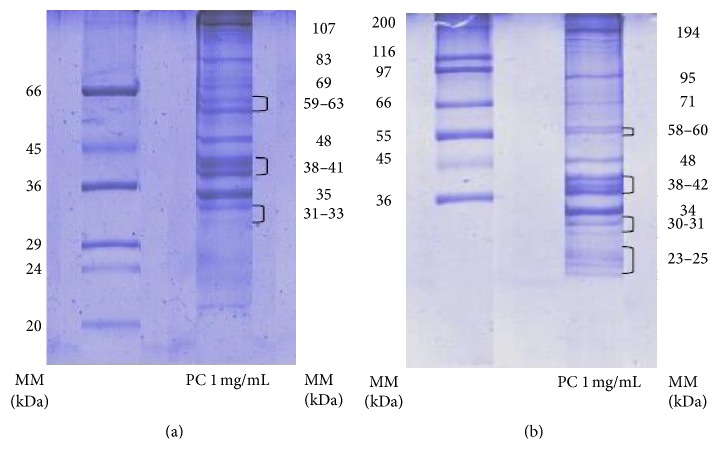
Protein fractions in the concentrate from SDS-PAGE with (a) a low molecular marker and 10% polyacrylamide and (b) a high molecular weight marker and 12% polyacrylamide, stained with Coomassie blue. PC: protein concentrate in a 1 mg/mL solution.

Protein patterns of the protein concentrate separated with 10 and 12% polyacrylamide (Figure 1) shows low and high molecular weight (MW) proteins. Bands between 31 and 66 kDa are visible; five of them show high intensity, with MW in the range of 33–48 kDa (Figure 1(a)); bands below 30 kDa separated with 12% polyacrylamide are shown in Figure 1(b). Proteins with MW below 23 kDa are not clearly observed and those present in high concentration are in the range of 31–48 kDa. Several bands >48 kDa are also visible, being the brightest for 58–60, 71, 95, and 194 kDa. The well-defined high molecular weight bands in Figure 1(b) correspond to 95 and 194 kDa, although their concentration is lower than those of the low molecular weight proteins. Several authors have characterized fish gelatin extracted from various fish species, showing similar protein patterns. Norziah et al. [26] characterized fish gelatin extracted from residues of surimi production and obtained two bands of similar molecular weights, identified as *α*-collagen (100 kDa) and *β*-collagen (200 kDa). The *β* component is formed when two simple collagen strands (*α* units) are cross-linked to each other by covalent bonds. Limpisophon et al. [14] also identified these two bands as *α* and *β* collagen in characterizing gelatin extracted from blue shark (*Prionace glauca*).

### 3.3. Solubility of the Protein Concentrates for Different pH

**Figure 2:**
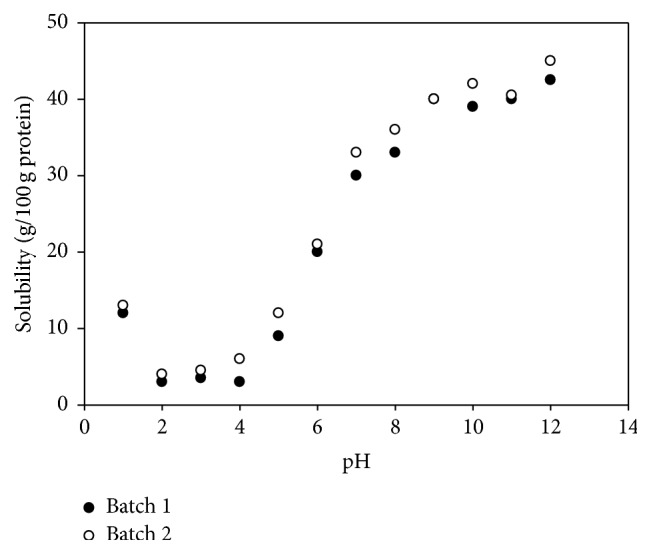
Solubility of the protein concentrates of Grouper skin batches for different pH.

Protein solubility depends on pH; above or below the isoelectric point (pI) the net charge is negative or positive, respectively, and water molecules can interact with these charges thereby contributing to solubility.  Figure 2 shows the change in solubility of the fish skin protein content of two batches as a function of pH in the range of 1 to 12. Both batches show a similar trend with no statistical difference, within the pI in the range of 2 to 4 pH units. As pH increases, the solubility of the protein concentrates increases because there are more negative charges enabling electrostatic repulsion with the solvent. The maximum solubility, 43.17%, occurred at pH 11 and 12. The purpose of knowing the solubility of the PC was to set the pH for film preparation. The pH 11.5 was chosen for film development considering the obtained data.

In other studies [27] collagen extracted from bigeye snapper skin with acid and pepsin exhibited the maximum solubility between pH values of 4 and 5, respectively. Also, Kittiphattanabawon et al. [28] observed that the maximum solubility of acid extracted collagen from big eye snapper skin was between pH 2 and pH 5. Likewise, the results in collagen obtained from chicken by-products show that the maximum collagen solubility was at pH 2.

### 3.4. Films Characteristics

Different formulations were tested to evaluate the effect of plasticizer type and concentration on film formation. Low molecular weight plasticizers incorporate easier into the protein matrix and in consequence they have a good performance on film formation [29]. The films of formulation 5PC-50G firmly adhered to the pan surface and broke up quickly. Formulation 5PC-75G produced films that could not be adequately formed because the plasticizer concentration was excessive; films remained moist for several days and could not be detached from the pan surface. Formulation 4PC-15G/15S produced films that were easily separated from the container surface but were less flexible than those with 5PC-75S. This behavior is attributed to the lower concentration of plasticizer. Films prepared 5PC-50S were brittle, stiff, and therefore unsuitable for subsequent analyses. Films with the best forming characteristics were 5PC-75S and 4PC-15G/15S. Film flexibility is mainly determined by protein-protein and protein-water interactions and may be controlled by the concentration and type of plasticizer, which reduces the intermolecular interactions between adjacent protein chains. As a consequence, chain mobility increases and films become flexible preventing rupture during handling and storage [30]. Our results show that it is possible to obtain films from Grouper fish skin proteins and that the incorporation of different kind of plasticizers into fish skin films resulted in more or less film flexibility and moisture.

Film formation has been proven with proteins extracted from different species of fish including Atlantic sardine (*Sardina pilchardus*) [31], red snapper (*Lutjanus vitta*) [6], and Nile tilapia (*Oreochromis niloticus*) [30].

### 3.5. Water Vapor Permeability

**Figure 3:**
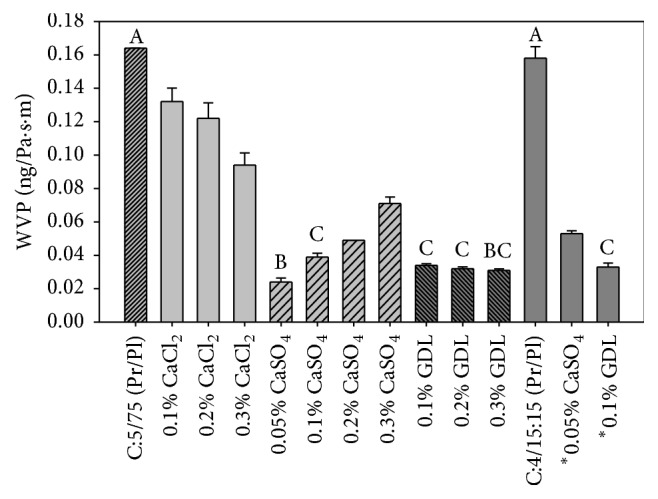
Water vapor permeability of unmodified and modified films prepared with formulations 5PC-75S and 4PC-15G/15S. Note: values in a column with different letters are significantly different at *p* < 0.05.

Figure 3 shows the effect of calcium salts and GDL on WVP of 5PC-75S and 4PC-15G/15S films. Each modifier produced a different effect. The addition of calcium chloride (CaCl_2_) decreased WVP of films; the higher the salt concentration, the lower the WVP. For 5PC-75S the WVP of the control film was reduced from 0.164 ng/Pa·s·m to 0.094 ng/Pa·s·m, that is, almost 42% when 0.3% CaCl_2_ was added. Calcium sulfate (CaSO_4_) had a greater impact on WVP than calcium chloride; the WVP of the control film was reduced to 0.070 ng/Pa·s·m, that is, around 57%, for 0.3% CaSO_4_. However, the effect of calcium sulfate was opposite to that of calcium chloride as lower concentrations of the former resulted in lower WVP. The addition of CaSO_4_ concentrations as low as 0.05% resulted in a WVP of 0.024 ng/Pa·s·m, which represents a reduction of almost 85% regarding that of the control. This suggests that the concentration of calcium chloride should be increased if a WVP similar to that with calcium sulfate wants to be obtained. Park et al. [11] reported statistically significant reductions in WVP of soy protein films modified with CaSO_4_. However, films modified with CaCl_2_ did not show significant decreases relative to control films. The authors explained that negative charges given by carboxyl groups (-COO^−^-) predominated on the protein chain and their interaction with divalent cations Ca^2+^ resulted in a more stable network. These ionic interactions not only reduce the mobility of protein segments but also increase their hydrophobicity as the interaction between Ca^2+^ and the negatively charged carboxyl groups prevents cations to interact with the water decreasing the solubility of proteins and thus WVP through the polymer.

The Hofmeister series can explain the difference between the effect caused by CaCl_2_ and CaSO_4_ on the WVP in our films as protein-protein interactions and protein crystallization are some of the physical behaviors that obey this series [32]. The series was originally developed as a measure of the efficiency of various anions to precipitate globular proteins. The effect of ions is usually related to their position in the series; SO_4_^2−^ > HPO_4_^2−^ > CH_3_COO^−^ > Cl^−^, which shows that the sulfate ion results in increased protein stability and lower solubility, as a result of greater protein-protein attraction, than the chloride ion [33]. The more significant effect caused by CaSO_4_ in comparison with CaCl_2_ on WVP of the protein films can be attributed to the fact that the SO_4_^2−^ ion is a better sequestrant of solvent water molecules and hence prevents the formation of hydrogen bonds on proteins surface. Therefore, effective protein-protein interactions occur that result in lower solubility and less water diffusion through the protein network.

The addition of glucono-*δ*-lactone (GDL) also reduced the WVP on 5PC-75S control films. WVP values were 0.034, 0.032, and 0.031 ng/Pa·s·m for 0.1, 0.2, and 0.3%, respectively, without being significantly different (Figure 3). GDL is a cyclic ester, gradually hydrolyzed in water to gluconic acid forms widely used in the food industry as acidulants [34]. The observed decrease in WVP of films modified with GDL may be due to an increased hydrophobicity and hence a reduction in solubility attributed to the charged carboxyl groups that adversely reduce the action of protons produced by GDL [11]. Therefore, the neutralized protein molecules can aggregate due to a decreased electrostatic repulsion and prevent carboxyl groups to interact with the water. The 5PC-75S films that exhibited the lower WVP were those modified with 0.05% CaSO_4_ and 0.1, 0.2, and 0.3% GDL. However, the WVP values of the latter were not significantly different. Figure 3 also shows the WVP of 4PC-15G/15S control films and films modified with 0.05% CaSO_4_ and 0.1% GDL, which were the modifiers and concentrations with the greater effect on the WVP of 5PC-75S films. The WVP of 4PC-15G/15S control films was 0.158 ng/Pa·s·m. Although it is lower than for 5PC-75S control films, there is not a significant difference between the two formulations because, even if the formulation 4PC-15G/15S had a lower concentration of protein, it also contains a smaller amount of plasticizers. Therefore, it is possible to assume that similar interactions occurred in films of both formulations. The WVP of the 4PC-15G/15S modified films significantly in comparison with their control. Calcium sulfate reduced WVP to 0.053 ng/Pa·s·m and GDL reduced it to 0.033 ng/Pa·s·m, which represent reductions of around 66 and 79%, respectively, regarding the control.

Comparing the results of all the films analyzed the formulation 5PC-75S with 0.05% CaSO_4_ and with different concentrations of GDL produced films with the best barrier against water vapor transmission, regardless of the concentration of protein and plasticizer. On the other side, the WVP of the unmodified films were of the same order of magnitude as those of protein films from other fish species. For example, the WVP of films made of skin proteins of Alaskan pink salmon was 0.169 ng/Pa·s·m [35], which is very similar to 0.164 ng/Pa·s·m for our 5PC-75S control film. However, our values for films modified with CaSO_4_ and GDL are lower by one or two orders of magnitude compared with films based on other protein concentrates, or synthetic polymers except polyester, which is still lower by one or two orders of magnitude, compared to the modified films in this study. These modifications are promising because modified films are expected to provide greater resistance to water transmission to the matrix they are covering, than films made up only of protein and plasticizer.

### 3.6. Oxygen Permeability

The stability of foods is affected by the presence or absence of oxygen. This gas affects the shelf life of foods because it participates in oxidation reactions, microorganism growth, changes in color, and respiration of fruits and vegetables [36]. Therefore, the oxygen permeability of protein films is essential for establishing their functionality as food protectants. Polymers containing groups that can self-associate by hydrogen or ionic bonds, such as proteins, produce films with excellent properties against oxygen permeability [37]. The average oxygen permeability of PC films of Grouper fish was 1.09 × 10^−17^ mol·mm/mm^2^·s·Pa. This corresponds to 3.27 × 10^−9^ cm^3^·cm/cm^2^·s·cmHg or 32.7 ± 0.79 barrers (1 barrer = 10^−10^ cm^3^·cm·cm^−2^·s^−1^·cmHg^−1^). The oxygen permeability of low density polyethylene at 298 K is 2.20 barrers [38]. This oxygen permeability is 15-fold lower than that of PC films of Grouper fish. Polymers with oxygen permeability below 38.9 cm^3^·*μ*m/m^2^·d·kPa (0.060 barrers) at 23°C are considered good barriers to oxygen [39]. In general, protein-based films are considered good oxygen barriers. Oxygen permeabilities reported for films of various proteins are lower than 38.9 cm^3^·*μ*m/m^2^·d·kPa [35].

### 3.7. Mechanical Properties

**Figure 4:**
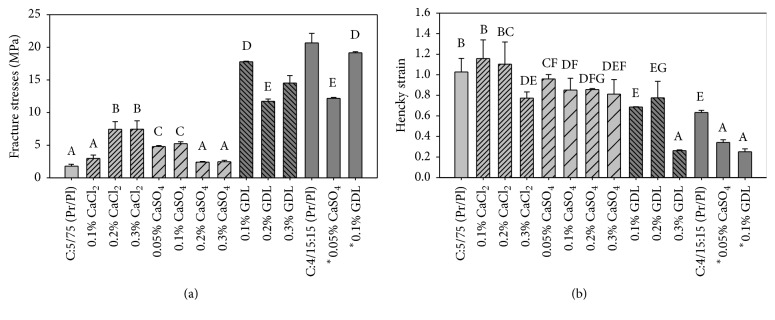
Fracture stresses (a) and Hencky strain (b) of films prepared with formulations 5PC-75S and 4PC-15G/15S.Note: values in a column with different letters are significantly different at *p* < 0.05.

The mechanical properties of the films provide an indication of their integrity under stresses associated with processing, handling, and storage.  Figure 4(a) shows the fracture stresses of unmodified (control) and modified 5PC-75S and 4PC-15G/15S films. The addition of 0.2 and 0.3% calcium chloride (CaCl_2_) to 5PC-75S films increased their fracture stress from 1.6 MPa, for the control, to around 7.3 MPa. The addition of 0.1% of this salt was not sufficient for obtaining a tensile fracture stress significantly different from that of the control and this also occurred for 0.2 and 0.3% CaSO_4_. On the contrary, the addition of 0.05 and 0.1% of this salt increased the fracture stress to about 5.0 MPa, which is lower than the increase with 0.2 and 0.3% calcium chloride. Therefore, low concentrations of calcium sulfate were sufficient to make films resistant to fracture upon stretching. Films modified with 0.1, 0.2, and 0.3% GDL showed fracture stresses between 11.7 and 17.8 MPa, which represents a significant increase over the 1.6 MPa for the control. These high values suggest the existence of greater protein interaction in modified films as compared with the control and those modified with calcium salts. According to Park et al. [11] GDL promotes protein aggregation because hydrophobicity is increased and solubility decreases, so films become more resistant but less flexible. The fracture stress of unmodified 4PC-15G/15S films was 20.7 MPa, which suggests a greater chain-chain interaction between proteins as the plasticizer concentration was lower than for unmodified 5PC-75S films. The addition of 0.05% CaSO_4_ and 0.1% GDL to 4PC-15G/15S films also improved the resistance of these materials to stretching. The fracture stresses of 5PC-75S films with 0.2% GDL and 4PC-15G/15S films with 0.05% CaSO_4_ were not significantly different. The same happened between 5PC-75S and 4PC-15G/15S films with 0.1 GDL.

The strain is given by a pure number, because it compares the shape of the material before and after deforming it. It is an important feature, because if a material can be stretched considerably before breaking up, this is an indication that it can withstand the applied load.  Figure 4(b) shows the trend of fracture strain, expressed as Hencky strain, for the two formulations of modified and unmodified films. In general, the addition of increasing concentrations of calcium salts and GDL resulted in significant reduction in fracture strain, with 0.3% GDL being the most noteworthy. The same trend was observed for 4PC-15G/15S films, but with a significant decrease in the control compared to the control of 5PC-75S films.

**Figure 5:**
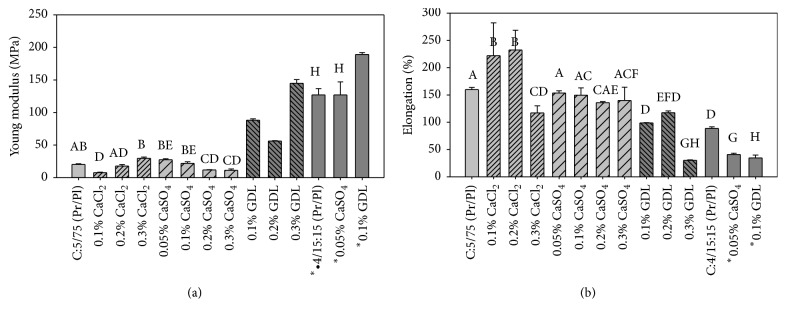
Young's modulus (a) and elongation percentage (b) of films prepared with formulations 5PC-75S and 4PC-15G/15S. Note: values in a column with different letters are significantly different at *p* < 0.05.

Young's modulus is the slope of the linear part of the stress-strain curve. It indicates the resistance of the material to deformation which is related to its stiffness.  Figure 5(a) shows Young's modulus for modified and unmodified 5PC-75S and 4PC-15G/15S films. Films modified with GDL were the most rigid of all the series according to their Young's modulus (Figure 5(a)) and fracture stress, regardless of the formulation. However, they did not withstand large deformations before breaking up because being more rigid due to increased interactions between polypeptides chains, they are more susceptible to deformation because of the reduced mobility of protein chains. This was the case, for example, for 4PC-15G/15S films modified with GDL and CaSO_4_. The fracture strains for 5PC-75S films modified with 0.1 and 0.2% CaCl_2_ were 1.16 and 1.10, respectively, because protein chains show more mobility.

The latter exhibited more resistance to deformation; 189 069 MPa for 0.1% GDL and 126 930 and 126 871 MPa for control and 0.05% CaSO_4_, respectively, without significant difference between both of them. These films also showed high fracture stresses, and this is again attributed to the lower concentration of plasticizer, which made them stiffer. In the case of 5PC-75S films modified with calcium salts, Young's modulus did not change considerably in comparison with the control. However, the film modified with 0.05% CaSO_4_, which had a lower WVP, exhibited the greatest Young's modulus, 27.4 MPa, in comparison with the control and films modified with calcium salts. This behavior confirms the presence of an increased cross-linking between proteins in the formulation. Therefore, these films were less elastic than those modified with calcium sulfate and calcium chloride.

Elongation is another way of expressing the flexibility of films to traction.  Figure 5(b) shows the percentage of elongation for the unmodified and modified formulations. 5PC-75S films can stretch over 100% of their original length, while a maximum elongation of about 232% was observed for films modified with 0.1 and 0.2% calcium chloride. The percentage of elongation for 4PC-15G/15S modified with 0.1% GDL and 0.05% CaSO_4_ was about 34.4%, while that for the control film was 88.5%. The 4PC-15G/15S and 5PC-75S films modified with GDL showed high fracture stress and Young's modulus, and therefore less percentage of elongation.

## 4. Conclusions

It was possible to produce films with proteins obtained from the skin of Grouper fish using an adequate proportion of PC and plasticizers. This means that proteins can form ordered three-dimensional networks capable of interacting with the plasticizer and water. The extraction yield of the protein concentrate from different batches is small. 5PC-75S films showed the best physical properties. Film formation was also possible with less protein, that is, 4% and a mixture of equal amounts of sorbitol and glycerol as plasticizers. The addition of different concentrations of calcium chloride, calcium sulfate, and GDL modifies mechanical and barrier properties differently and to different extents. Permeability to oxygen was not detected over a period of 24 hours. This result could be convenient to retard chemical, physical, and microbiological degradation in foods and offers an alternative to the use of a biodegradable packaging. Protein films of Grouper fish skin were better barriers to water vapor and oxygen compared with protein films from other natural sources. Traction tests evidenced the greater resistance of films modified with GDL, which were less deformable and resistant to physical changes during handling. In general, the 5PC-75S formulation exhibits a high stretching capacity than 4PC-15G/15S. Films modified with GDL and CaSO_4_ represent a significant advance in film technology based on proteins. The lack of oxygen permeability significantly reduced WVP and acceptable mechanical properties compared to other proteins are attractive properties for the materials studied here.

## Figures and Tables

**Table 1:** Films formulations.

Identification	Protein concentrate (%)	Plasticizer
5PC-50G	5	50% glycerol
5PC-75G	5	75% glycerol
5PC-50S	5	50% sorbitol
5PC-75S	5	75% sorbitol
4PC-15G/15S	4	15% glycerol + 15% sorbitol

**Table 2:** Modified films.

Identification	Protein concentrate (%)	Plasticizer	Modified salts
5PC-75S	5	75% sorbitol	0.1% CaCl_2_
5PC-75S	5	75% sorbitol	0.2% CaCl_2_
5PC-75S	5	75% sorbitol	0.3% CaCl_2_
5PC-75S	5	75% sorbitol	0.05% CaSO_4_
5PC-75S	5	75% sorbitol	0.1% CaSO_4_
5PC-75S	5	75% sorbitol	0.2% CaSO_4_
5PC-75S	5	75% sorbitol	0.3% CaSO_4_
5PC-75S	5	75% sorbitol	0.1% GDL
5PC-75S	5	75% sorbitol	0.2% GDL
5PC-75S	5	75% sorbitol	0.3% GDL
4PC-15G/15S	4	15% glycerol + 15% sorbitol	0.05% CaSO_4_
4PC-15G/15S	4	15% glycerol + 15% sorbitol	0.1% GDL

## References

[1] Petersen K., Nielsen P. V., Bertelsen G. (1999). Potential of biobased materials for food packaging. *Trends in Food Science and Technology*.

[2] Bourlieu C., Guillard V., Vallès-Pamiès B., Guilbert S., Gontard N. (2009). Edible moisture barriers: how to assess of their potential and limits in food products shelf-life extension?. *Critical Reviews in Food Science and Nutrition*.

[3] Krochta J., Balwin E., Nísperos-Carriedo M. (1994). *Edible Coatings and Films to Improve Food Quality*.

[4] Avella M., De Vlieger J. J., Errico M. E., Fischer S., Vacca P., Volpe M. G. (2005). Biodegradable starch/clay nanocomposite films for food packaging applications. *Food Chemistry*.

[5] Tharanathan R. N. (2003). Biodegradable films and composite coatings: past, present and future. *Trends in Food Science & Technology*.

[6] Jongjareonrak A., Benjakul S., Visessanguan W., Prodpran T., Tanaka M. (2006). Characterization of edible films from skin gelatin of brownstripe red snapper and bigeye snapper. *Food Hydrocolloids*.

[7] Guilbert S., Cuq B., Gontard N. (1997). Recent innovations in edible and/or biodegradable packaging materials. *Food Additives & Contaminants*.

[8] Foegeding E., Lanier T. C., Hultin H. O., Fennema O. R. (1996). Characteristics of edible muscle tissue. *Food Chemistry*.

[9] Castañeda P. K. (2011). *Desarrolllo y evaluación de propiedades de películas proteínicas de pesquerías de la subclase Elasmobranchii [Ph.D. thesis]*.

[10] Pires C., Ramos C., Teixeira G. (2011). Characterization of biodegradable films prepared with hake proteins and thyme oil. *Journal of Food Engineering*.

[11] Park S. K., Rhee C. O., Bae D. H., Hettiarachchy N. S. (2001). Mechanical properties and water-vapor permeability of soy-protein films affected by calcium salts and glucono-*δ*-lactone. *Journal of Agricultural and Food Chemistry*.

[12] Zactiti E. M., Kieckbusch T. G. (2006). Potassium sorbate permeability in biodegradable alginate films: effect of the antimicrobial agent concentration and crosslinking degree. *Journal of Food Engineering*.

[13] CONAPESCA Anuario estadístico de acuacultura y pesca. http://www.conapesca.sagarpa.gob.mx/wb/cona/anuario_2008.

[14] Limpisophon K., Tanaka M., Weng W., Abe S., Osako K. (2009). Characterization of gelatin films prepared from under-utilized blue shark (*Prionace glauca*) skin. *Food Hydrocolloids*.

[15] Hoque M. S., Benjakul S., Prodpran T. (2011). Properties of film from cuttlefish (*Sepia pharaonis*) skin gelatin incorporated with cinnamon, clove and star anise extracts. *Food Hydrocolloids*.

[16] AOAC (1995). *Official Methods of Analysis*.

[17] Batista I. (1999). Recovery of proteins from fish waste products by alkaline extraction. *European Food Research and Technology*.

[18] Lowry O. H., Rosebrough N. J., Farr A. L., Randall R. J. (1951). Protein measurement with the Folin phenol reagent. *The Journal of Biological Chemistry*.

[19] Laemmli U. K. (1970). Cleavage of structural proteins during the assembly of the head of bacteriophage T4. *Nature*.

[20] Saeed M., Cheryan M. (1988). Sunflower protein concentrates and isolates low in polyphenols and phytate. *Journal of Food Science*.

[21] Sobral P. J. A., Menegalli F. C., Hubinger M. D., Roques M. A. (2001). Mechanical, water vapor barrier and thermal properties of gelatin based edible films. *Food Hydrocolloids*.

[22] Vanin F. M., Sobral P. J. A., Menegalli F. C., Carvalho R. A., Habitante A. M. Q. B. (2005). Effects of plasticizers and their concentrations on thermal and functional properties of gelatin-based films. *Food Hydrocolloids*.

[23] ASTM E 96-95 Standard test method for water vapor transmission of materials.

[24] ASTM (2003). Standard test method for determining gas permeability characteristics of plastic film and sheeting. *ASTM*.

[25] ASTM D 882 (2002). Standard test method for tensile properties of thin plastic sheeting. *1997 Annual Book of ASTM Standards*.

[26] Norziah M. H., Al-Hassan A., Khairulnizam A. B., Mordi M. N., Norita M. (2009). Characterization of fish gelatin from surimi processing wastes: thermal analysis and effect of transglutaminase on gel properties. *Food Hydrocolloids*.

[27] Jongjareonrak A., Benjakul S., Visessanguan W., Tanaka M. (2005). Isolation and characterization of collagen from bigeye snapper (*Priacanthus macracanthus*) skin. *Journal of the Science of Food and Agriculture*.

[28] Kittiphattanabawon P., Benjakul S., Visessanguan W., Nagai T., Tanaka M. (2005). Characterisation of acid-soluble collagen from skin and bone of bigeye snapper (*Priacanthus tayenus*). *Food Chemistry*.

[29] Bourtoom T., Chinnan M. S., Jantawat P., Sanguandeekul R. (2006). Effect of plasticizer type and concentration on the properties of edible film from water-soluble fish proteins in surimi wash-water. *Food Science and Technology International*.

[30] Paschoalick T. M., Garcia F. T., Sobral P. J. A., Habitante A. M. Q. B. (2003). Characterization of some functional properties of edible films based on muscle proteins of Nile Tilapia. *Food Hydrocolloids*.

[31] Cuq B., Gontard N., Cuq J.-L., Guilbert S. (1997). Selected functional properties of fish myofibrillar protein-based films as affected by hydrophilic plasticizers. *Journal of Agricultural and Food Chemistry*.

[32] Zhang Y., Cremer P. S. (2006). Interactions between macromolecules and ions: the Hofmeister series. *Current Opinion in Chemical Biology*.

[33] Curtis R. A., Lue L. (2006). A molecular approach to bioseparations: protein-protein and protein-salt interactions. *Chemical Engineering Science*.

[34] Trop M., Kushelevsky A. (1985). The reaction of glucono delta lactone with proteins. *Journal of Dairy Science*.

[35] Chiou B.-S., Avena-Bustillos R. J., Bechtel P. J., Imam S. H., Glenn G. M., Orts W. J. (2009). Effects of drying temperature on barrier and mechanical properties of cold-water fish gelatin films. *Journal of Food Engineering*.

[36] Robertson G. L. (2006). *Food Packaging, Principles and Practice*.

[37] Damodaran S., Paraf A. D. (1997). Food proteins. An overview. *Food Proteins and Their Applications*.

[38] Permeability Coefficient of Common Polymers (Plastics) http://www.faybutler.com/pdf_files/HowHoseMaterialsAffectGas3.pdf.

[39] Hong S.-I., Krochta J. M. (2006). Oxygen barrier performance of whey-protein-coated plastic films as affected by temperature, relative humidity, base film and protein type. *Journal of Food Engineering*.

